# Recent progress in realizing novel one-dimensional polymorphs via nanotube encapsulation

**DOI:** 10.1186/s40580-024-00460-3

**Published:** 2024-12-04

**Authors:** Yangjin Lee, Uje Choi, Kwanpyo Kim, Alex Zettl

**Affiliations:** 1 Department of Energy Science, Sungkyunkwan University, Suwon, 16419 Korea; 2 Department of Physics, Yonsei University, Seoul, 03722 Korea; 3 Department of Physics, University of California at Berkeley, Berkeley, CA 94720 USA; 4 Materials Sciences Division, Lawrence Berkeley National Laboratory, Berkeley, CA 94720 USA; 5 Kavli Energy NanoSciences Institute, University of California at Berkeley, Berkeley, CA 94720 USA

**Keywords:** Nanotube, Encapsulation, Geometrical confinement, One-dimensional materials, Atomic chain

## Abstract

Encapsulation of various materials inside nanotubes has emerged as an effective method in nanotechnology that facilitates the formation of novel one-dimensional (1D) structures and enhances their functionality. Because of the effects of geometrical confinement and electronic interactions with host nanotubes, encapsulated materials often exhibit low-dimensional polymorphic structures that differ from their bulk forms. These polymorphs exhibit unique properties, including altered electrical, optical, and magnetic behaviors, making them promising candidates for applications in electronics, energy storage, spintronics, and quantum devices. This review explores recent advancements in the encapsulation of a wide range of materials such as organic molecules, elemental substances, metal halides, metal chalcogenides, and other complex compounds. In particular, we focus on novel polymorphs formed through the geometrical confinement effect within the nanotubes. The atomic structure, other key properties, and potential applications of these encapsulated materials are discussed, highlighting the impact of nanotube encapsulation on their functionalities.

**Graphical Abstract:**

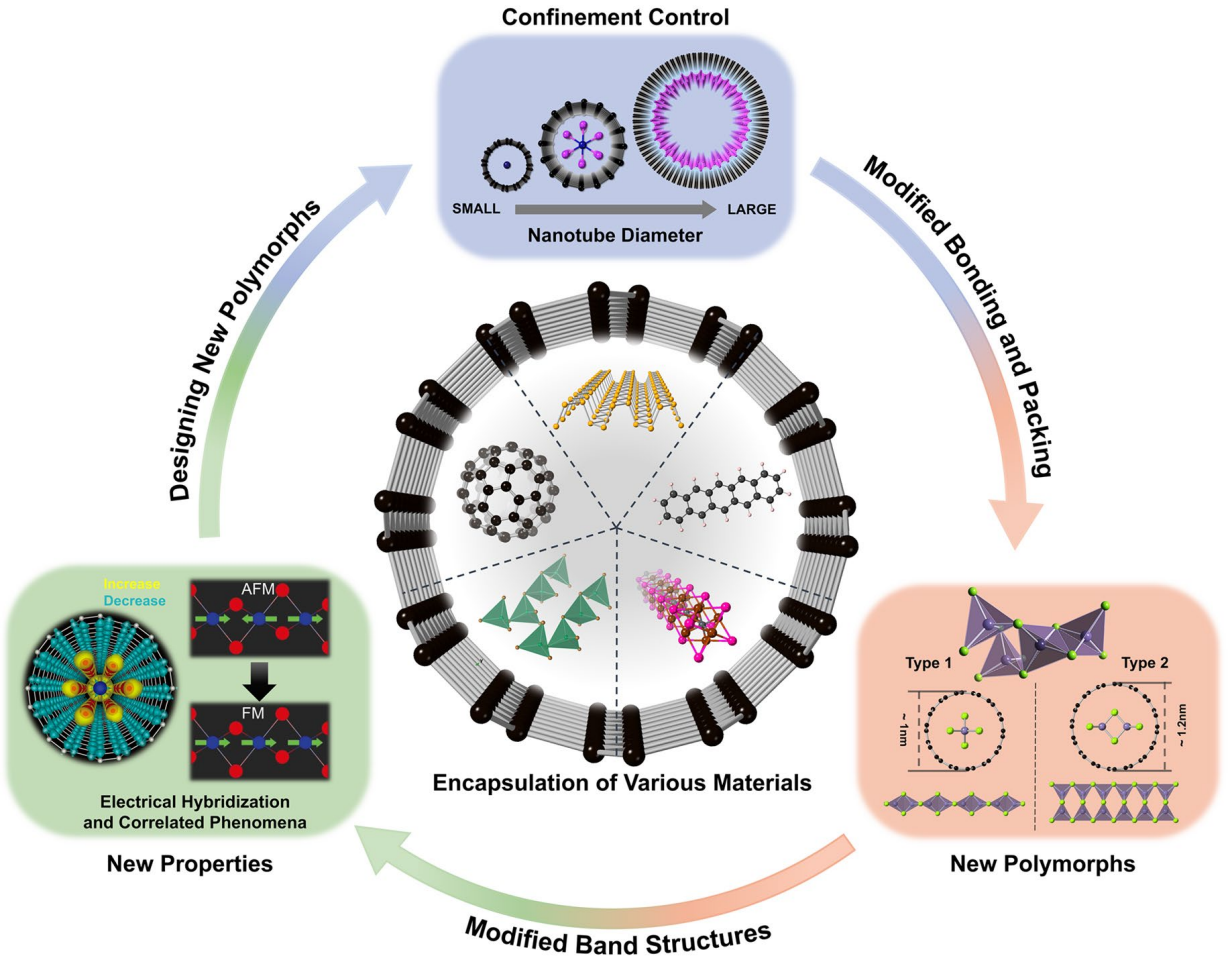

## Introduction

Carbon nanotubes (CNTs) and boron nitride nanotubes (BNNTs) are exemplary one-dimensional (1D) nanomaterials that have played a pivotal role in enabling atomic-scale materials characterization and driving various scientific breakthroughs [1–14]. One of the key features of nanotubes is their internal hollow space, which ranges from sub-nanometer to several hundred nanometers in diameter, offering the potential to host other materials through encapsulation. Over the past few decades, encapsulation of various materials inside nanotubes has emerged as a critical method in nanotechnology, enabling the formation of novel heterostructures and enhancing material functionalities [15–23].

Both single-walled and multi-walled nanotubes are effective for encapsulation. Typically, multi-walled nanotubes are employed, simply because they are more readily available. Here the innermost nanotube serves as a geometrical boundary for the encapsulation process, and the behavior of the encapsulated material and the resulting structure are highly dependent on the diameter of this innermost tube. Bulk materials, without significant modifications, can be inserted into large-diameter nanotubes. However, when the diameter reaches the sub nanometer or nanometer scale, strong geometrical confinement effects occur. These effects often lead to the formation of new packing structures and altered bonding configurations, as observed in various encapsulated materials [18, 24–30].

The properties of encapsulated materials are also fundamentally transformed by reduced dimensionality and constrained environments, resulting in modified electrical, optical, and magnetic behaviors [30–37]. These altered properties have the potential to drive advancements in the fields of electronics, energy storage, catalysis, and quantum devices [38–46]. The confinement effect of the nanotubes allows the stabilization of materials with new structures and compositions that are difficult to achieve in unconfined bulk states [21, 30, 47–51]. Confinement via encapsulation is particularly beneficial for stabilizing materials that are highly sensitive to environmental factors such as moisture, light, and oxygen, allowing researchers to explore systems that would otherwise be challenging to study [25, 27, 29, 30, 52–55].

In this review, we summarize recent advances in the encapsulation of various materials inside nanotubes, focusing on the impact of nanoscale confinement on structural formation and property modification. We cover a broad range of materials, including carbon polymorphs, elemental substances, metal halides, metal chalcogenides, perovskites, and metal carbides, each of which undergoes a remarkable structural transformation upon confinement within the nanotubes. Furthermore, we highlight the emerging properties of these new polymorphs and explore their potential applications, illustrating the transformative role of nanotube encapsulation in the advancement of next-generation nanomaterials and technologies.

## Nanotube encapsulation for novel polymorphic structures

Various synthesis methods have been successfully utilized for nanotube encapsulation of materials such as metals, metal oxides, halides, chalcogenides, and organic compounds [16, 23, 24, 28–30, 36, 49, 56–62]. Among the various encapsulation methods, the vapor transport method is the most widely used, offering high efficiency and yields for a wide range of materials [18, 23, 30, 55, 63, 64]. Liquid-based encapsulation is another exemplary method [40, 52, 65]. Bottom-up one-pot syntheses have also been used with a limited number of materials [56, 66, 67].

Several advanced characterization methods have been employed to explore the structure and properties of materials encapsulated within nanotubes [37, 68–76]. Among these, transmission electron microscopy (TEM) and scanning TEM (STEM) are crucial for identifying new polymorphic structures in encapsulated materials [23, 26, 28, 54, 55, 77, 78]. In particular, aberration-corrected TEM/STEM enables direct atomic-scale observations, allowing researchers to explore novel structures formed within nanotubes. In addition, in situ TEM techniques provide real-time observations of structural changes in encapsulated materials under external stimuli, such as electron beam irradiation, heating/cooling, and electrical biasing, offering valuable insights into the dynamic behaviors within the TEM environment [67, 68, 79–91].

Furthermore, when combined with spectroscopic techniques such as energy-dispersive X-ray spectroscopy (EDS) and electron energy loss spectroscopy (EELS), these methods go beyond structural analysis. They enable detailed exploration of the elemental composition, electronic states, and optical properties at the single-atom level, even within confined spaces [55, 77, 78, 87, 92–97]. This powerful combination of atomic-resolution imaging and spectroscopy is essential for understanding the unique behaviors and transformations of encapsulated materials induced by nanoscale confinement.

**Fig. 1:**
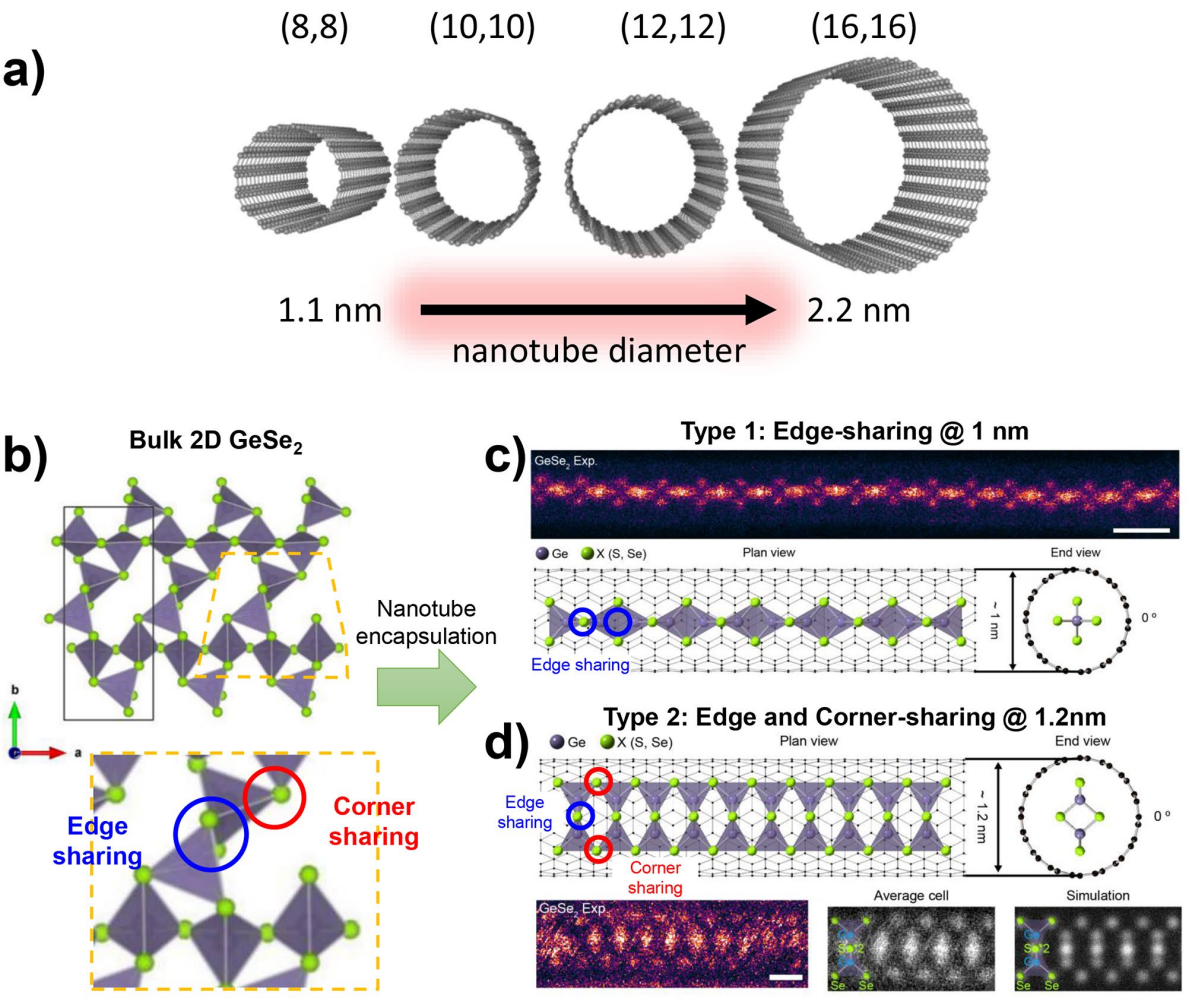
Variation in the connectivity of encapsulated materials with nanotube diameters. (**a**) Variation in nanotube diameter with different chirality. (b-d) Tuning the GeX_4_ tetrahedrons connection mode via nanotube encapsulation with different diameter of nanotubes. (**b**) Atomic model of single layer of 2D GeX_2_ structure. GeX_4_ tetrahedrons are composed of edge-sharing and corner-sharing with 1:1 ratio. (**c**) Type-1 1D GeX_2_ single-chain encapsulated within nanotube of diameter 1 nm. GeX_2_ chain is composed of edge shared GeX_4_ tetrahedrons. (**d**) Type-2 1D GeX_2_ single-chain encapsulated within nanotube of diameter 1.2 nm. The GeX_2_ chain is composed of edge and corner shared GeX_4_ tetrahedrons. (**b**-**d**) Reproduced with permission from Ref. [28], Copyright © 2023, American Chemical Society

Inner diameters of multiwall CNTs and BNNTs range from sub nanometers to tens of nanometers. In principle, both the diameter and chirality of the nanotube could affect the structure of the encapsulated material **(**Fig. 1a**)**. In practice, the nanotube inner diameter is the dominant tuning parameter. The typically weak bonding between the encapsulated species and the nanotube lessens the geometrical impact of the nanotube chirality. On the other hand, aside from geometrical confinement, the surrounding nanotube can serve as a charge donor or acceptor to the encapsulated species. In this case the metallicity of the nanotube is important. BNNTs are insulating independent of diameter and chirality, whereas for single-walled CNTs diameter and chirality do affect metallicity. Multi-walled CNTs are typically metallic throughout. Hence, for both multi-wall CNT and BNNT encapsulation, nanotube chirality is not a key tuning parameter. When a material is filled into a nanotube, the spatial constraints of the nanotube size impose firm spatial restrictions on the encapsulated material, often shifting the energy landscape and resulting in unique atomic arrangements and phase behaviors that are not observed in the bulk material [26–30, 48, 49, 98, 99]. Table 1 summarizes the key examples of the formation of new structures depending on the inner diameter of the host nanotubes.

**Table 1:** Summary of various materials encapsulated inside nanotubes

Material	Tube diameter (nm)	Outcomes	Ref
Linear C chain	0.71	Long linear carbon single chain was stabilized	[21]
P	~ 1	Tetrahedra P4 molecule, zigzag chain, and zigzag ladder structures were investigated	[52, 99]
	1 ~ 1.4	1D square columnar phosphorus was observed	[27]
	4.1	Zigzag black phosphorus ribbons were stabilized	[27]
	5 ~ 8	Ring-shape phosphorus structure was investigated	[25]
S	0.6 ~ 0.7	Linear and zigzag single sulfur chain were investigated	[133]
Se	1	A double helix Se structure was stabilized within nanotube, and a single helix Se structure was formed by the electron beam	[134]
Te	0.8~	Te atomic chains were stabilized inside nanotube from multiple to single-chain limit	[38, 93]
I	0.75 ~ 1.4	Single, double, triple helical chains, and trimerized single chain were investigated	[130–132]
Eu	0.76 ~ 1.7	1D metallic Eu atomic wires were synthesized with high filling yields	[47]
MoTe/WTe	1.3	Mo/W Te single atomic wire was synthesized	[64, 171]
SnSe	0.7 ~ 1.3	Linear dipole chain, zig-zag, 2 × 1, 2 × 2 cubic, and MoSe-like SnSe structure were investigated	[26]
Kr@C_60_	1.4	After coalescence of Kr@C_60_ by heat treatment, 1D gas-like Kr inside nanotube was formed	[87]
Ge/Si X_2_ (S, Se)	1	1D edge shared tetrahedral single chain of Ge and Si dichalcogenides were stabilized	[28, 29]
	1.2	1D edge and corner shared tetrahedral single chain of Ge and Si dichalcogenides were stabilized	[28, 29]
Cr/V X_3_ (Cl, Br, I)	1.3	1D single chain of face shared octahedral Cr and V trihalides were stabilized	[30]
Nb/V/Ti Te_3_	~ 1	Nb/V/Ti Te_3_ single chains were synthesized via nanotube encapsulation	[49]
NbSe_3_	1.2 ~ 3.8	Single to multiple chain of NbSe_3_ were synthesized, and charge induced torsional waves in single-chain NbSe_3_ were investigated	[23]
HfTe_3_	1.2 ~ 3.8	Single to multiple chain of HfTe_3_ were synthesized. Spiraling double and triple chain were observed	[60]
TaTe_y_	1 ~ 10	TaTe_2_, TaTe_3_, and TaTe_y_ superlattice structures were synthesized	[179]
Hf_2_Te_9_	1.1	Segmented Hf_2_Te_9_ linear chain was stabilized	[48]
CsPbBr_3_/CsSnI_3_	1.2 ~ 1.6	The smallest perovskites were encapsulated within nanotube	[54, 55]
W/Mo _2_C	0.8 ~ 2	< 110 > preferred W_2_C and Mo_2_C nanowires were investigated	[183]
High entropy compounds	1.26	High entropy compounds containing various elements were synthesized	[97]
Mo_8_S_8_Cl_11_	1~	Mo_8_S_8_Cl_11_ nanoribbons, composed of Mo_4_S_4_ clusters connected by Cl atoms, were synthesized inside nanotube	[51]

A striking example of the transformative potential of nanotube encapsulation is the behavior of germanium dichalcogenides (GeX_2_; X = S, or Se) [28]. In its bulk form, GeX_2_ exhibits a two-dimensional (2D) layered structure, but when encapsulated within a nanotube, it undergoes significant structural modifications owing to geometrical confinement. In the single-layer form of GeX_2_, GeX_4_ tetrahedra are connected via edge and corner-sharing modes in a 1:1 ratio **(**Fig. 1b**)**. However, upon encapsulation in nanotubes, the arrangement of these tetrahedra changes dramatically depending on the diameter of the nanotube. At a diameter of approximately 1 nm, where only one tetrahedron could fit, the tetrahedra align into a 1D chain in an edge-sharing configuration **(**Fig. 1c**)**. When the nanotube diameter increases slightly to approximately 1.2 nm, the 1D chain structure persists, but the connection mode changes. In this case, the GeX_4_ tetrahedra still exhibit edge-sharing but are also adopt corner-sharing connections **(**Fig. 1d**)**. These unique 1D polymorphs are not observed in bulk GeX_2_, highlighting how even small variations in the nanotube diameter can precisely control the structural arrangement of the encapsulated materials.

The ability of nanotube encapsulation to induce novel structural configurations through precise control of nanotube diameter underscores its potential for creating materials with tailored properties [26–29, 38, 60, 100]. This offers new possibilities for designing functional nanomaterials with unique behaviors, especially when combined with the ability to stabilize new compositions and phases that are challenging to achieve in bulk. Nanotube encapsulation also provides a valuable platform for exploring systems that are highly sensitive to environmental factors, such as moisture, light, and oxygen, enabling detailed studies that are difficult under ambient conditions [25, 27, 29, 30, 52–55]. Examples of these materials, including perovskites, metal halides, and metal chalcogenides, are presented in the following sections.

## Examples of materials encapsulated inside nanotubes

The following sections provide examples of a variety of materials successfully encapsulated in nanotubes, focusing on the structural organization revealed by TEM/STEM based characterizations, such as imaging and spectroscopic analysis. Among the many examples, we highlight materials that form novel nanostructures within nanotubes that are difficult to realize in their bulk form. These advanced characterization techniques provide insights into the morphology, arrangement, and interaction of encapsulated materials at the atomic and molecular levels, thereby elucidating how confinement within nanotubes influences their properties and potential applications.

### Carbon allotropes (fullerene and other allotropes)

**Fig. 2:**
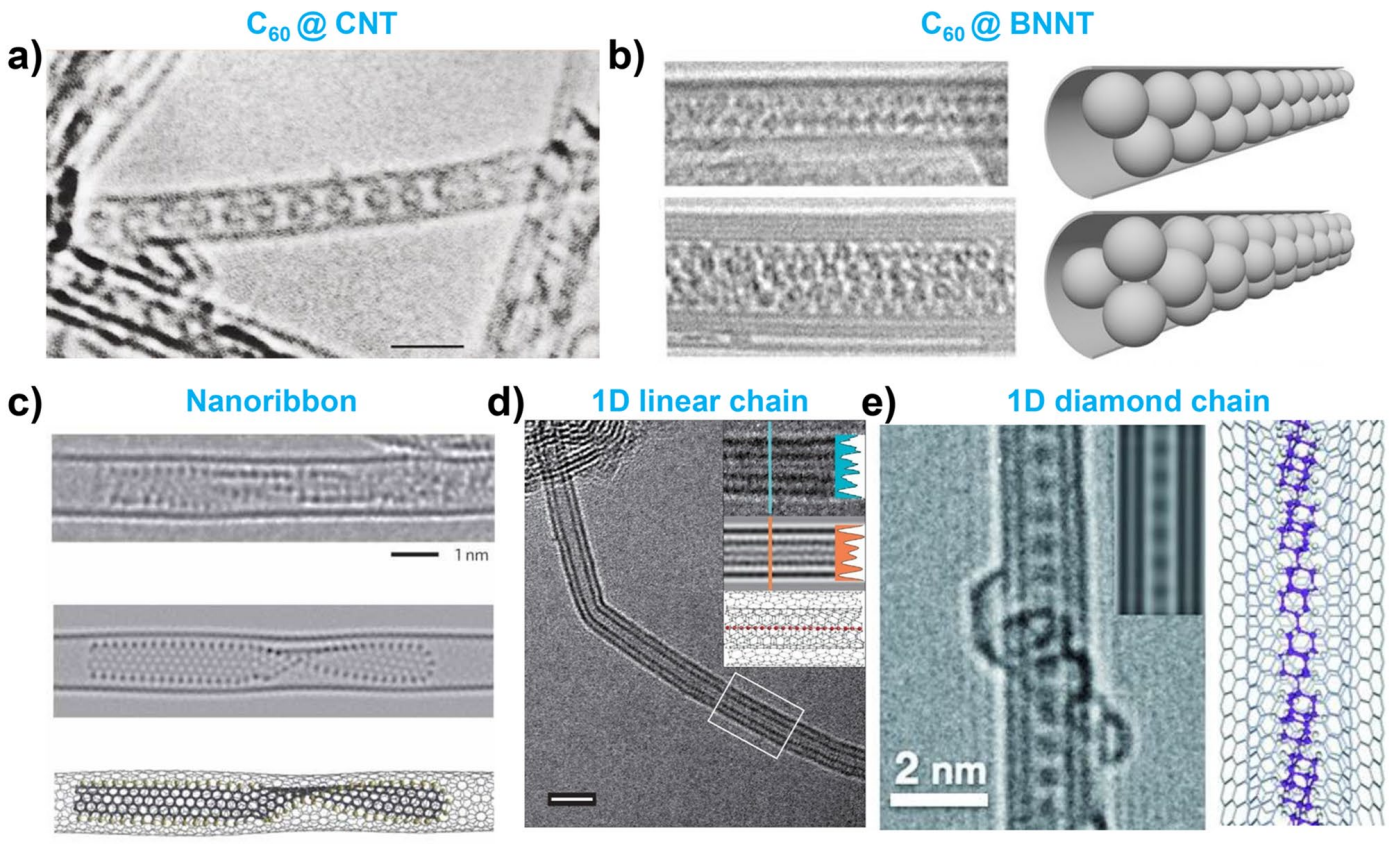
Carbon allotropes encapsulated inside nanotubes. (**a**) HRTEM image of C_60_ encapsulation within CNT. Reproduced with permission from ref. [17], copyright © 1998, Springer Nature. (**b**) TEM images of staggered C_60_ nanowire and rotated triangle C_60_ nanowire inside BNNT of different diameters. Reproduced with permission from ref. [18], Copyright © 2003, The American Association for the Advancement of Science. (**c**) Graphene nanoribbon encapsulated inside a CNT. Reproduced with permission from ref. [113], Copyright © 2011, Springer Nature. (**d**) 1D long linear carbon chain (LLCC) encapsulated inside CNT. Reproduced with permission from ref. [21], copyright © 2016, Springer Nature. (**e**) 1D diamond chain encapsulation inside CNT. Reproduced with permission from ref. [123], Copyright © 2015, John Wiley and Sons

Carbon allotropes, particularly fullerenes, are among the earliest and most widely studied materials encapsulated inside nanotubes. The encapsulation of fullerenes forms unique 1D structures, including “peapod” configurations, where the C_60_ molecules are arranged in a linear chain within the nanotube. In 1998, Smith et al. first reported the encapsulation of C_60_ inside CNTs [17]. The C_60_@CNT structure was identified from CNTs synthesized using pulsed laser vaporization. They confirmed that C_60_ and CNT were stabilized in nanotubes with a diameter of 1.3 nm with a van der Waals gap of 0.3 nm **(**Fig. 2a**)**. This pioneering work demonstrated the potential of nanotubes as nanoscale containers for encapsulating molecular structures and laid the foundation for further research on the encapsulation of various materials within nanotubes.

With the successful synthesis of BNNTs [2, 101, 102], research efforts have expanded to incorporate C_60_ into BNNTs. Mickelson et al. reported a linear chain of C_60_ encapsulated within ultranarrow BNNTs, which was similar to the results for CNTs [18]. Their study revealed that the packing arrangement of C_60_ was highly dependent on the inner diameter of the nanotubes (d), leading to the formation of various new C_60_ nanostructures. Specifically, the following structural changes were observed with increasing nanotube diameters: for d = 2.0 nm, a staggered C_60_ nanowire forms; for d = 2.8 nm, a rotated triangular (3-corkscrew) C_60_ nanowire appears; for d = 3.3 nm, a 7-corkscrew C_60_ nanowire appears; and for d = 4.0 nm, disordered stacking of C_60_ molecules occurs **(**Fig. 2b**)**. Finally, when the nanotube diameter is sufficiently large, the boundary conditions imposed by the nanotube walls become insignificant, allowing the C_60_ molecules to stack in a more conventional crystalline close-packed manner. This study highlights the crucial role of the nanotube diameter in determining the structural organization of encapsulated materials, providing a valuable method for tailoring nanoscale properties through the precise control of nanotube dimensions.

Consequently, numerous studies have been conducted to fill and characterize various types of fullerenes, including endofullerenes, within nanotubes [22, 32, 79, 80, 83, 84, 87, 103–111]. In particular, research has focused on modifying the structure of encapsulated fullerenes such as C_60_ and C_70_ to other forms of fullerenes using an electron beam inside the TEM [79, 80, 83, 84, 103, 112]. These studies have demonstrated the ability to manipulate fullerene molecules, leading to observations of fullerene dimerization, transformation into CNT, graphene nanoribbons **(**Fig. 2c**)** and molecular dynamic behaviors [79, 80, 83, 84, 113, 114].

Another significant achievement in carbon allotrope encapsulation involves the synthesis of long linear carbon chains (LLCCs). Carbyne, the sp1 hybridized 1D linear carbon chain, is predicted to exhibit unique physical and chemical properties [115]. However, owing to its extreme instability under ambient conditions, synthesizing long carbyne has been known to be highly challenging [116, 117]. In 2016, Shi et al. successfully demonstrated the synthesis of LLCCs encapsulated inside double-walled CNTs (DWCNTs) for the first time **(**Fig. 2d**)** [21]. Confinement within the nanotube provides a stable environment that shields the LLCC from external degradation and reactivity, thus allowing researchers to explore its novel properties [118, 119].

In addition, studies have been conducted not only to encapsulate various carbon containing compounds into CNTs, but also to convert them, post encapsulation, into other carbon structures [34, 63, 71, 120–126]. 1D linear-chain diamond structures **(**Fig. 2e**)** were synthesized using diamantine dicarboxylic acid as a precursor [122, 123]. The sublimed precursors undergo dehalogenation to form linear-chain diamantane polymers inside the CNTs. Similarly, graphene nanoribbons have been obtained through the incorporation of aromatic molecules or sulfur-containing molecules [120, 121].

### Elemental materials within the nanotube

In this section, we briefly explore the encapsulation of elemental materials, excluding carbon, inside nanotubes. Typically, nanotubes can be efficiently filled with elemental materials at low sublimation (or melting) temperatures by using a high-vacuum heating process. Various elemental substances, including low melting point elements such as phosphorus (P), arsenic (As), antimony (Sb), iodine (I), sulfur (S), selenium (Se), and tellurium (Te), as well as metallic substances such as europium (Eu), are directly encapsulated within nanotubes through this simple heating process [25, 27, 38, 47, 52, 53, 93, 99, 127–134]. A host of high melting point metallic elements, including magnetic iron (Fe) and cobalt (Co), have also been successfully encapsulated [59, 91].

**Fig. 3:**
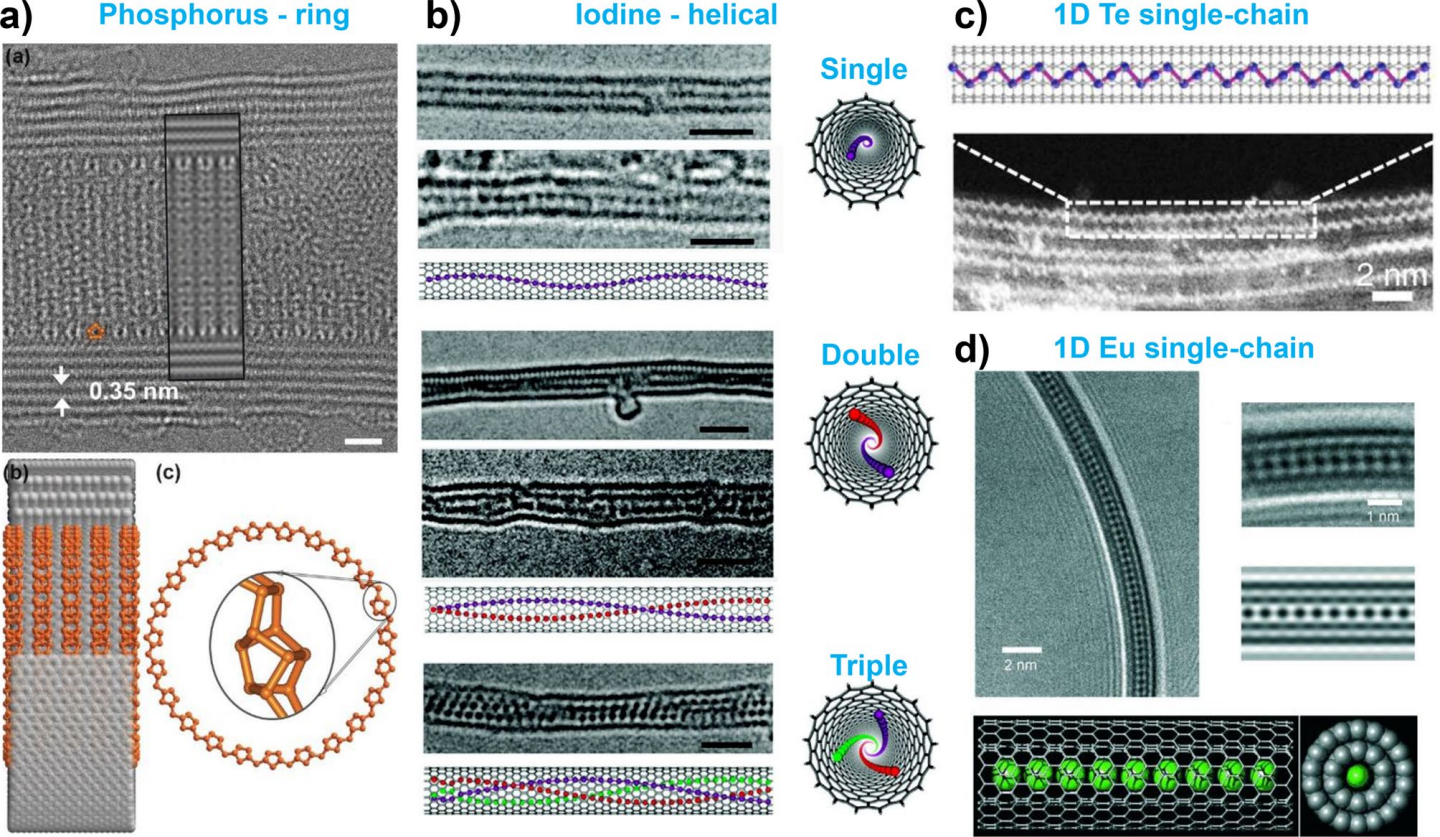
Elemental encapsulation within the nanotubes. (**a**) HRTEM image of ring shape phosphorus structure encapsulated inside CNTs with the structural models. Reproduced with permission from ref. [25], Copyright © 2017, John Wiley and Sons. (**b**) HRTEM images of single, double, and triple helical iodine chains inside CNTs. Reproduced with permission from ref. [131], Copyright © 2007, American Chemical Society. (**c**) Atomic model and HR-STEM image of Te single-chain encapsulation within the nanotubes. Reproduced with permission from ref. [38], Copyright © 2020, Springer Nature. (**d**) HRTEM image of 1D Eu single-chain encapsulation inside CNT with the structural models. Reproduced with permission from ref. [47], Copyright © 2009, John Wiley and Sons

Phosphorus is of particular interest because of its multiple allotropes, including white, red, black, and violet phosphorus, as well as its potential undiscovered forms [135–141]. When confined within nanotubes, phosphorus exhibits a unique structure. Zhang et al. reported the formation of ring-shaped phosphorus nanostructures consisting of alternating P8 and P2 units within CNTs with diameters ranging from 5 to 8 nm **(**Fig. 3a**)** [25]. This was the first experimental confirmation of ring-shaped phosphorus, a structure that had been previously predicted only using theoretical models [138, 142]. Subsequently, Zhang et al. observed square columnar phosphorus in nanotubes with a diameter of 1 nm and zigzag black phosphorus ribbons within nanotubes with a dimeter of 4 nm [27]. Hart et al. observed P_4_ tetrahedral molecular chain, zigzag chain, and double-stranded chain structures within the nanotubes [52, 99]. In addition, they expended their research to other pnictogens such as arsenic (As) and antimony (Sb) and observed similar structures [53, 99]. Research on phosphorus with different structural configurations is actively ongoing, and it is predicted that unique forms, such as helical phosphorus, will be achievable within nanotube confinements [127–129, 142].

Iodine, a stable and heavy halogen that exists as a solid at room temperature, can be efficiently encapsulated inside nanotubes owing to its relatively low sublimation temperature. Upon heating, iodine easily vaporizes and fills the nanotubes with a high yield [130–132]. Experimentally, single, double, and triple helical iodine chains have been observed, and the critical nanotube diameter for the formation of triple chains is 1.45 nm **(**Fig. 3b**)** [131]. As the diameter of the nanotubes increases, the formation of quadruple or more chains cannot be observed, and iodine tends to adopt a bulk orthorhombic crystal structure. In the single-chain iodine structures, transitions between equidistant and trimerized chains have been observed, which were attributed to charge transfer interactions between the iodine chain and the nanotube [132].

For chalcogens (S, Se, and Te), single chains have been successfully encapsulated within the narrow diameter of CNTs (less than 1 nm). Linear and zigzag chains of sulfur have been identified, and helical chain structures of Se and Te have been investigated [38, 93, 133, 134]. Bulk tellurium is composed of chains bound by van der Waals forces, and when encapsulated in nanotubes (either CNT or BNNT), it retains the same chain structure, with only the number of chains varying depending on the nanotube diameter **(**Fig. 3c**)** [38]. This observation highlights the ability of nanotube confinement to isolate bulk materials composed of chain structures down to the single-chain limit.

Kitaura et al. reported the encapsulation of a 1D europium (Eu) metal chain that was directly sublimated and crystallized inside nanotubes [47]. A single chain of Eu was encapsulated within nanotubes with a diameter of 0.76 nm **(**Fig. 3d**)**, while double and four Eu chains were formed inside nanotubes with diameters of 1.06 nm and 1.54 nm, respectively. The interatomic distance between neighboring Eu atoms in the single-chain structure (0.467 nm) was found to be elongated compared to that in the bulk crystal (0.397 nm), possibly because of charge-transfer interaction between the Eu atoms and CNTs.

For other elemental metals with high sublimation temperatures, a multi-step sequential chemical reaction method was employed to encapsulate them within the nanotubes. In this method, nanotubes are pre-filled with metal-organic precursors, oxides, halides, or metallofullerenes, which are then subjected to a reduction process to form pure metal structures inside the nanotubes [16, 59, 143–146]. This method has been successfully applied to metals such as Fe, Au, Ag, Pt, In, Co, Re, and others [16, 59, 143, 145–147]. The diameters of the nanotubes pre-filled with the precursors are relatively large, resulting in the formation of metal nanowires or particles rather than single atomic chains. In some cases, metals are encapsulated within the nanotubes during the nanotube synthesis [56, 66, 67].

### Metal-halides encapsulation inside nanotubes

The encapsulation of metal halides (MX, MX_2_, MX_3_, and other stoichiometries, where M represents all types of metals and X represents a halogen, such as Cl, Br, or I) within nanotubes leads to the formation of unique nanostructures with fascinating electrical, optical, and magnetic properties. Typically, halide materials are highly sensitive to ambient exposure such as moisture, light, and oxygen, which often limit their stability and practical applications [148–150]. By encapsulating these materials inside nanotubes, not only can new structures be formed due to the geometrical confinement effects, but their stability can also be dramatically enhanced, protecting them from degradation.

Many kinds of metal halides inside nanotubes have been investigated. These metal halides include alkali metal halides such as KI, NaI, CsI, CsCl and LiI [19, 24, 77, 78, 151–155]; alkaline earth metal halides such as BaI_2_ [156]; transition metal halides such as AgI, CuI, AgCl, AgClI, HgI_2_, NiI_2_, CoI_2_, CdCl_2_, CrI_3_, CrBr_3_, CrCl_3_, VI_3_, VBr_3_, VCl_3_, and ZnCl_4_ [20, 24, 30, 73, 76, 153, 157–159]; as well as post-transition metal halides such as PbI_2_ [37, 70, 160–162]. Rare earth and actinide halides have also been investigated, including CeI, LaI_2_, CeI_3_, CeCl_3_, LaI_3_, LaCl_3_, NdCl_3_, SmCl_3_, EuCl_3_, TbCl_3_, GdCl_3_, ErCl_3_, GdI_3_, YbCl_3_, and ThCl_6_ [24, 35, 96, 163–166]. In Addition, halides of noble metals and metalloids, such as AuCl_3_, BiI_3_, and BiCl_3_ have also been encapsulated [167, 168]. Among the numerous examples of metal halides encapsulated within nanotubes, we highlight a few notable cases.

**Fig. 4:**
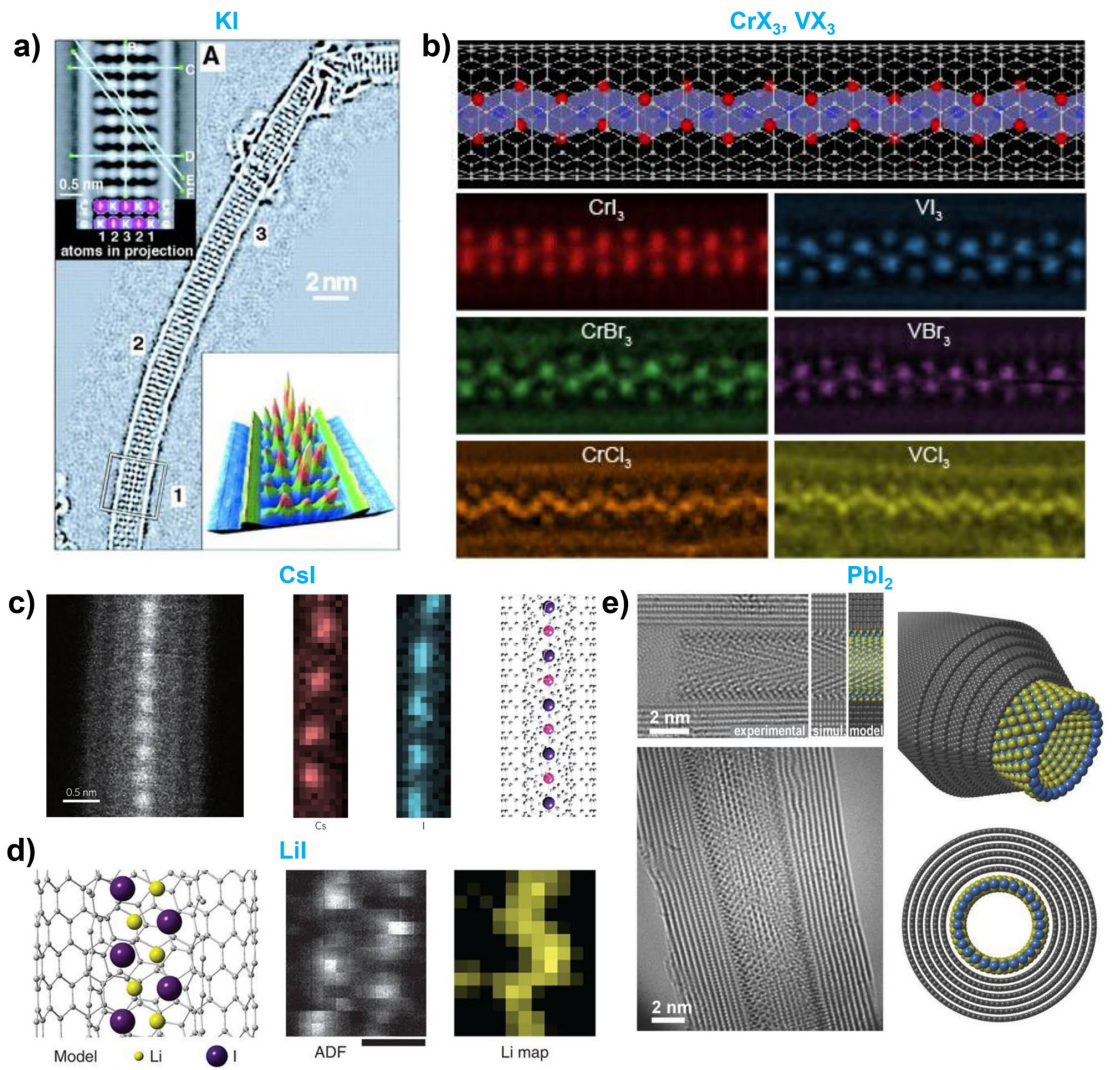
Metal halides encapsulation inside nanotube. (**a**) 3 × 3 KI encapsulation inside CNT of diameter 1.6 nm. Reproduced with permission from ref. [19], Copyright © 2000, The American Association for the Advancement of Science. (**b**) HR-STEM images of 1D chromium and vanadium trihalides (MX_3_) encapsulated within the nanotube. Reproduced with permission from ref. [30], Copyright © 2023, John Wiley and Sons. (**c**) Atomic resolution STEM imaging and EELS mapping of CsI encapsulation within CNTs. Reproduced with permission from ref. [77], Copyright © 2014, Springer Nature. (**d**) Atomic resolution STEM EELS-characterization of LiI within CNTs. Reproduced with permission from ref. [78], Copyright © 2015, Springer Nature. (**e**) HR-TEM images of PbI_2_ nanotubes within the CNTs. Reproduced with permission from ref. [161], Copyright © 2013, John Wiley and Sons

Potassium iodide (KI) is one of the earliest studied metal halides for encapsulation in nanotubes. In 2000, Meyer et al. and Sloan et al. conducted pioneering studies on the diameter-dependent structures of KI encapsulated within carbon nanotubes (CNTs) using a molten phase filling method [24]. Sloan et al. observed a 2 × 2 crystal structure of KI within 1.4 nm diameter CNTs, while Meyer et al. found a 3 × 3 KI crystal structure inside 1.6 nm diameter CNTs **(**Fig. 4a**)** [19, 151]. Depending on the atomic positions within the nanotube-encapsulated KI, the coordination is reduced from the bulk 6:6 coordination, where each ion is surrounded by six nearest neighbors, to 5:5 or 4:4, based on the location of the atoms. In the two-layer structure confined within a 1.4 nm CNT, only 4:4 coordination is observed. However, for the three-layer KI crystals within a 1.6 nm CNT, the central row of atoms retains the bulk-like 6:6 coordination, whereas the face and edge atoms exhibit reduced 5:5 and 4:4 coordination because of the spatial constraints imposed by the nanotube [19, 24, 151].

Since these pioneering studies, numerous investigations have been conducted to encapsulate various metal halides within nanotubes, including 1D helical CoI_2_, lanthanide halide crystals, and other metal halides [20, 24, 70, 96, 152, 153, 155, 156, 158, 160, 163, 164, 169, 170]. Recently, Lee et al. successfully encapsulated 1D magnetic chains of CrX_3_ and VX_3_ within CNTs of diameter 1.1 nm **(**Fig. 4b**)** [30]. Their study revealed that octahedral units, which typically form 2D layered structures via edge sharing in bulk CrX_3_ and VX_3_, instead form 1D chain structures inside the nanotubes by adopting a face-sharing configuration. This geometrical transformation is attributed to the spatial confinement imposed by the nanotubes, which forces the octahedrons to form a linear arrangement. Furthermore, the study demonstrated that these 1D chains were stabilized by charge transfer between the CNTs and encapsulated materials, enhancing their overall structural stability and changing their magnetic properties.

A truly 1D atomic single chain of CsI has been successfully encapsulated within carbon nanotubes [77]. Using atomic-level EELS characterization, individual atoms in the CsI chain were precisely identified, confirming the arrangement of the Cs and I atoms along the nanotube axis **(**Fig. 4c**)**. Similarly, other alkali-halide encapsulated CNTs, such as NaI, CsCl, and LiI, have been synthesized and characterized **(**Fig. 4d**)** [78]. The direct imaging of Li is typically challenging owing to its low atomic number and high reactivity. With the stabilization of Li within the nanotubes, single-atom EELS mapping was successfully achieved, enabling the precise identification of Li atoms within the confined nanotube environment.

In addition to the 1D chain and nanowire structures, the nanotube structure of metal halide materials has been stabilized inside CNTs with a wide diameter. Cabana et al. synthesized PbI_2_ nanotube structures of diameter 3.5–7.5 nm encapsulated inside the MWCNT **(**Fig. 4e**)** [161]. They found that the formation of the PbI_2_ nanotubes depends strongly on the diameter of the host CNTs. Other metal halide nanotubes, including ZnI_2_, TbCl_3_, CeI_3_, CeCl_3_, BiI_3_, BiCl_3_, and GdI_3_, have been synthesized within MWCNTs using similar methods [165, 166, 168].

### Metal-chalcogenides encapsulation inside nanotubes

**Fig. 5:**
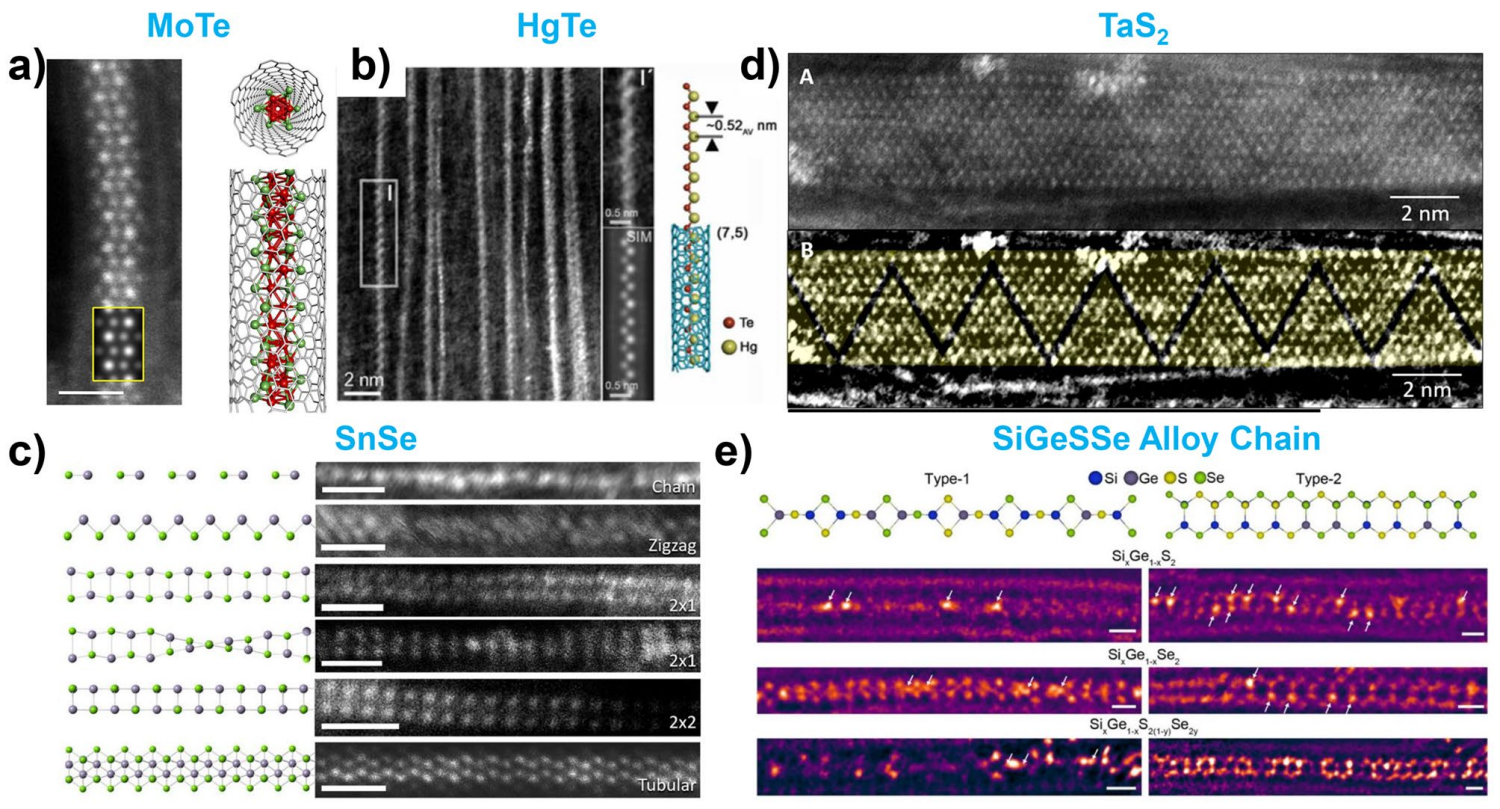
Transition metal monochalcogenides (MX) and dichalcognides (MX_2_) encapsulation within the nanotubes. (**a**) HR-STEM image of MoTe single-wire with atomic model. Reproduced with permission from ref. [64], Copyright © 2019, American Chemical Society. (**b**) HR-STEM image of zigzag HgTe single chain inside CNTs. Reproduced with permission from ref. [100], Copyright © 2022, American Chemical Society. (**c**) HR-STEM images of various SnSe structures within the nanotube with corresponding atomic model. Reproduced with permission from ref. [26], Copyright © 2019, American Chemical Society. (**d**) ADF-STEM image and its filtered version (bottom) of periodic superstructure in TaS_2_ nanoribbons. The superstructure is identified as defect line arrays, with lines of missing S atoms. Reproduced with permission from ref. [82], Copyright © 2021, American Chemical Society. (**e**) ADF-STEM images of SiGeSSe alloy single chain encapsulation inside CNT. Reproduced with permission from ref. [29], Copyright © 2024, American Chemical Society

**Fig. 6:**
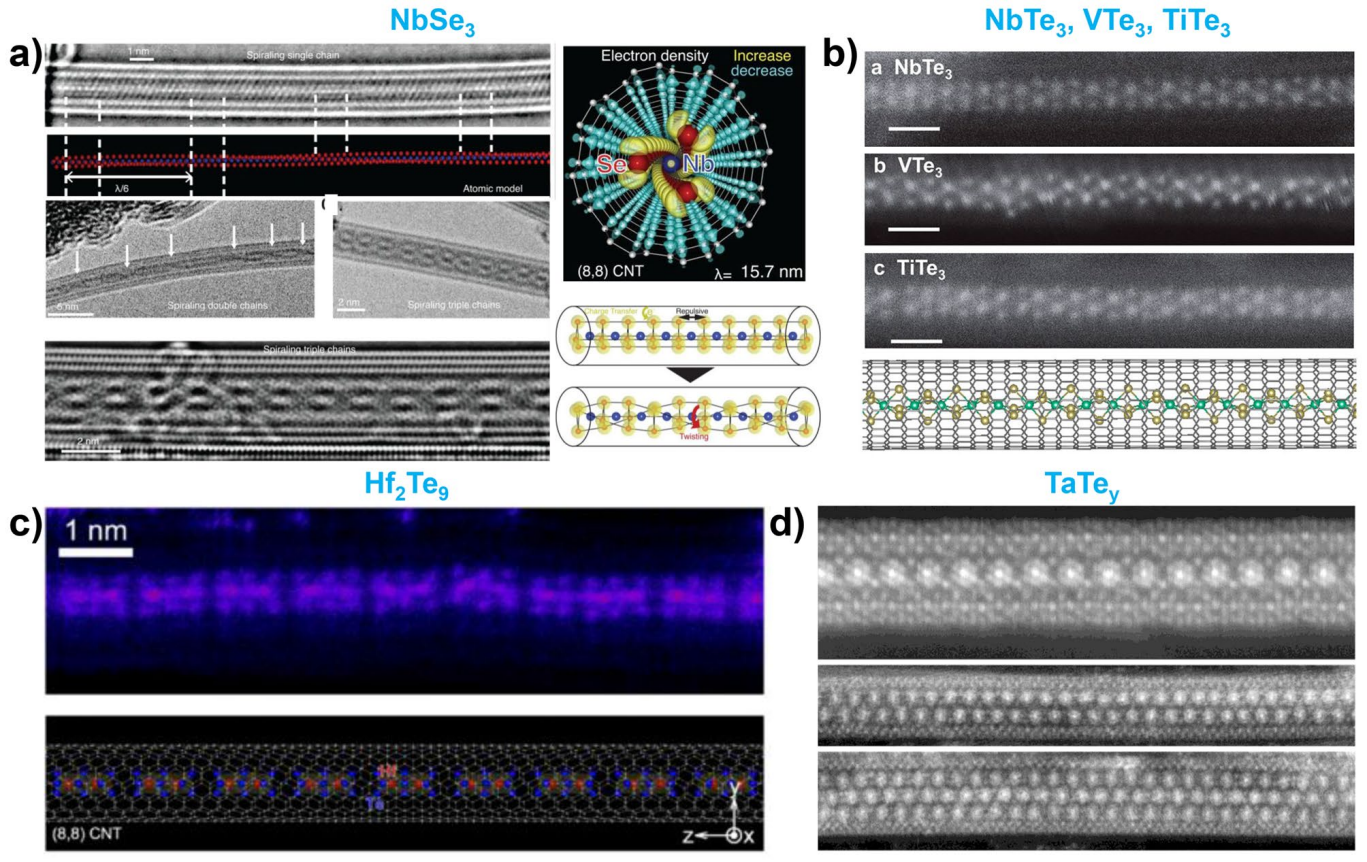
Transition metal trichalcognides (MX_3_) and other chalcogenides with different stoichiometry. (**a**) NbSe_3_ encapsulation inside CNT and BNNT. Charge induced torsional motion was observed. Reproduced with permission from ref. [23], Copyright © 2018, The American Association for the Advancement of Science. (**b**) HR-STEM images of NbTe_3_, VTe_3_, and TiTe_3_ encapsulation within the CNT. Reproduced with permission from ref. [49], Copyright © 2021, American Chemical Society. (**c**) HR-STEM image of segmented molecule structure of Hf_2_Te_9_ inside CNT. Reproduced with permission from ref. [48], Copyright © 2020, American Physical Society. (**d**) Moire like TaTe_y_ superstructure encapsulation within the CNT. Reproduced with permission from ref. [179], Copyright © 2022, American Chemical Society

In this section, we discuss metal chalcogenides (MX, MX_2_, MX_3_, and other stoichiometries, where M represents all types of metals and X represents a chalcogens such as S, Se, and Te) encapsulated within nanotubes. As mentioned previously, encapsulation within nanotubes enables the formation of structures and compositions that are challenging to achieve in bulk materials. By leveraging this feature, a wide range of metal chalcogenides with diverse combinations of metals and chalcogens were stabilized within the nanotubes, as shown in Figs. 5 and 6.

Starting with MX, molybdenum telluride (MoTe) in the form of 1D single nanowires has been stabilized inside CNTs **(**Fig. 5a**)** [64]. Partially oxidized MoTe_2_ is used as the precursor and it is proposed that MoO_x_ oxidizes MoTe_2_ to MoTe and TeO_2_. Similarly, transition metal swapped tungsten telluride (WTe) with the same structure has also been successfully stabilized with a high filling yield using WO_x_ with a Te precursor [171]. Mercury telluride (HgTe) was stabilized within nanotubes in two distinct structures, zigzag **(**Fig. 5b**)** and tubular, depending on the diameter of the CNTs [72, 100, 172]. This variation in structural formation is strongly influenced by the confinement effects imposed by the nanotube diameter [172, 173]. Slade et al. explored the structural diversity of tin selenide (SnSe) when encapsulated in nanotubes of different diameters [26]. They observed the various encapsulated SnSe structures, including linear, zigzag, spiral, MoSe-like, and even the previously explored 2 × 2 cubic structure within nanotubes of diameter 0.7–1.3 nm **(**Fig. 5c**)** [174]. In addition, materials such as indium selenide (InSe), tin telluride (SnTe), lead telluride (PbTe) and germanium telluride (GeTe) have been successfully stabilized within nanotubes, further demonstrating the potential of nanotube encapsulation to form unique structures [81, 85, 94, 98, 175].

For MX_2_ compounds, many metal dichalcogenides have been encapsulated within nanotubes to create highly ordered nanostructures. Transition-metal dichalcogenides usually have a 2D layered structure in the bulk; therefore, they prefer to form nanoribbon structures within nanotubes. MoS_2_, WS_2_, ReS_2_, TaS_2_, HfTe_2_, and TaTe_2_ nanoribbons are stabilized inside nanotubes using methods such as CVT or subsequent reaction processing [82, 86, 176–179]. Nanoribbons formed within the nanotubes generally maintain the same structure as the bulk 2D layer. However, structural transitions can occur during TEM/STEM characterization because of the influence of the electron beam, and these transformed structures can also be stabilized within the nanotube. For example, TaS_2_ nanoribbons grown within nanotubes exhibit structural transformation into a periodic superstructure with an ordered array of linear defects by an electron beam, which is not typically observed in bulk samples **(**Fig. 5d**)** [82]. For HfTe_2_ nanoribbons, a change from the metallic phase to the semiconducting phase was also observed [86]. NbSe_2_ nanotubes were found to form flattened structures within chemically driven self-pressurized carbon nanotubes, further demonstrating the diversity of structural formations achieved through nanotube confinement [180].

For non-transition metal dichalcogenides, Lee et al. investigated the 1D tetrahedral chain structures of Si and Ge dichalcogenides (SiX_2_ and GeX_2_) encapsulated within nanotubes [28, 29]. They demonstrated that the nanotube diameter significantly influences the formation of these chain structures. For nanotubes with diameters smaller than 1 nm, 1D tetrahedral chains are formed via edge-sharing. In nanotubes with a diameter of 1.2 nm, 1D tetrahedra are formed through both edge- and corner-sharing. Furthermore, they expanded the compositional and structural diversity of these 1D chains by implementing alloyed chains composed of Si, Ge, S, and Se mixtures within the nanotubes **(**Fig. 5e**)** [29]. This approach enables the synthesis of alloyed 1D chains with tunable compositions and properties, demonstrating the versatility of nanotube encapsulation for controlling material structures at the nanoscale.

Transition-metal trichalcogenides (MX_3_) are representative examples of MX_3_ material that have been successfully encapsulated in nanotubes [23, 49, 60, 68, 179]. These materials typically consist of quasi-1D chain crystals in bulk form. Encapsulating MX₃ materials within nanotubes allows for the isolation and manipulation of these quasi-1D materials in the few-chain and single-chain limit, allowing for the exploration of the effects of dimensionality reduction. Pham et al. synthesized single- or few-chains NbSe_3_ encapsulated within the nanotubes (both CNTs and BNNTs) [23]. Their study revealed that as the thickness (chain number) of NbSe_3_ decreased, charge-induced structural torsion waves occurred in the NbSe_3_ chains, which were not observed in the bulk structure **(**Fig. 6a**)**. This dimensionality reduction introduces new physics that can only be observed at single- or few-chain level [68]. Stonemeyer et al. synthesized single to multiple chains of HfTe_3_ inside the CNTs [60]. Unlike NbSe_3_, which has a trigonal prismatic (TP) structure, HfTe_3_ has a trigonal anti-prismatic (TAP) structure. They found that single chains of HfTe_3_ did not exhibit torsional waves, whereas double and triple chains exhibited the interchain spiral structures. Moreover, Stonemeyer et al. expanded the set of materials studied by synthesizing and stabilizing multiple and single chains of NbTe_3_, VTe_3_, TiTe_3_, and TaTe_3_ inside nanotubes, which are materials that are not stable in their bulk form **(**Fig. 6b**)** [49, 179].

Finally, other metal chalcogenides with different stoichiometries such as Sb_2_S_3_, Sb_2_Se_3_, Sb_2_Te_3_, Hf_2_Te_9_, Zr_11_Te_50_, and TaTe_y_ were studied [48, 50, 74, 75, 179, 181]. Confinement within the nanotubes allows the formation of compositions and structures that do not typically occur in bulk materials. Pham et al. discovered a segmented linear chain of Hf_2_Te_9_, which is a novel structure that has not been previously explored **(**Fig. 6c**)** [48]. The segmented chains were bonded end-to-end by van der Waals forces, similar to the 1D structure of C_60_. Pelz et al. identified a Zr_11_Te_50_ complex structure encapsulated within CNTs, and determined that it consisted of a combination of 8 ZrTe_5_ chains, 3 ZrTe_2_ chains, and 4 single Te chains [50]. Stonemeyer et al. synthesized various Ta-Te structures, including TaTe_2_, TaTe_3_, and TaTe_y_ superlattice structures. The encapsulated TaTe_y_ superlattice structures exhibited Moiré-like patterns with different configurations, increasing in width from single to double and triple patterns **(**Fig. 6d**)** [179]. Quasi-1D van der Waals crystals of antimony trichalcogenides (Sb_2_S_3_, Sb_2_Se_3_, and Sb_2_Te_3_) were successfully encapsulated within nanotubes, ranging from nanowire bundles to single chains. Both crystalline and amorphous forms of these materials have been encapsulated in CNTs and BNNTs [75]. The formation of either crystalline or amorphous structures is influenced by the diameter of the nanotubes, which plays a critical role in determining their structural configuration [74]. Additionally, these materials have been reported to undergo structural transitions from crystalline to amorphous under e-beam irradiation, highlighting their dynamic behavior under external stimuli [181].

### Other materials inside the nanotubes

In this section, we briefly discuss other materials, not included in the abovementioned material families, that have been successfully encapsulated within nanotubes. Various materials have been explored, including perovskites, noble gas, metal carbides, high-entropy compounds, metal oxide, and other compounds [51, 54, 55, 58, 61, 87, 97, 152, 182–186].

**Fig. 7:**
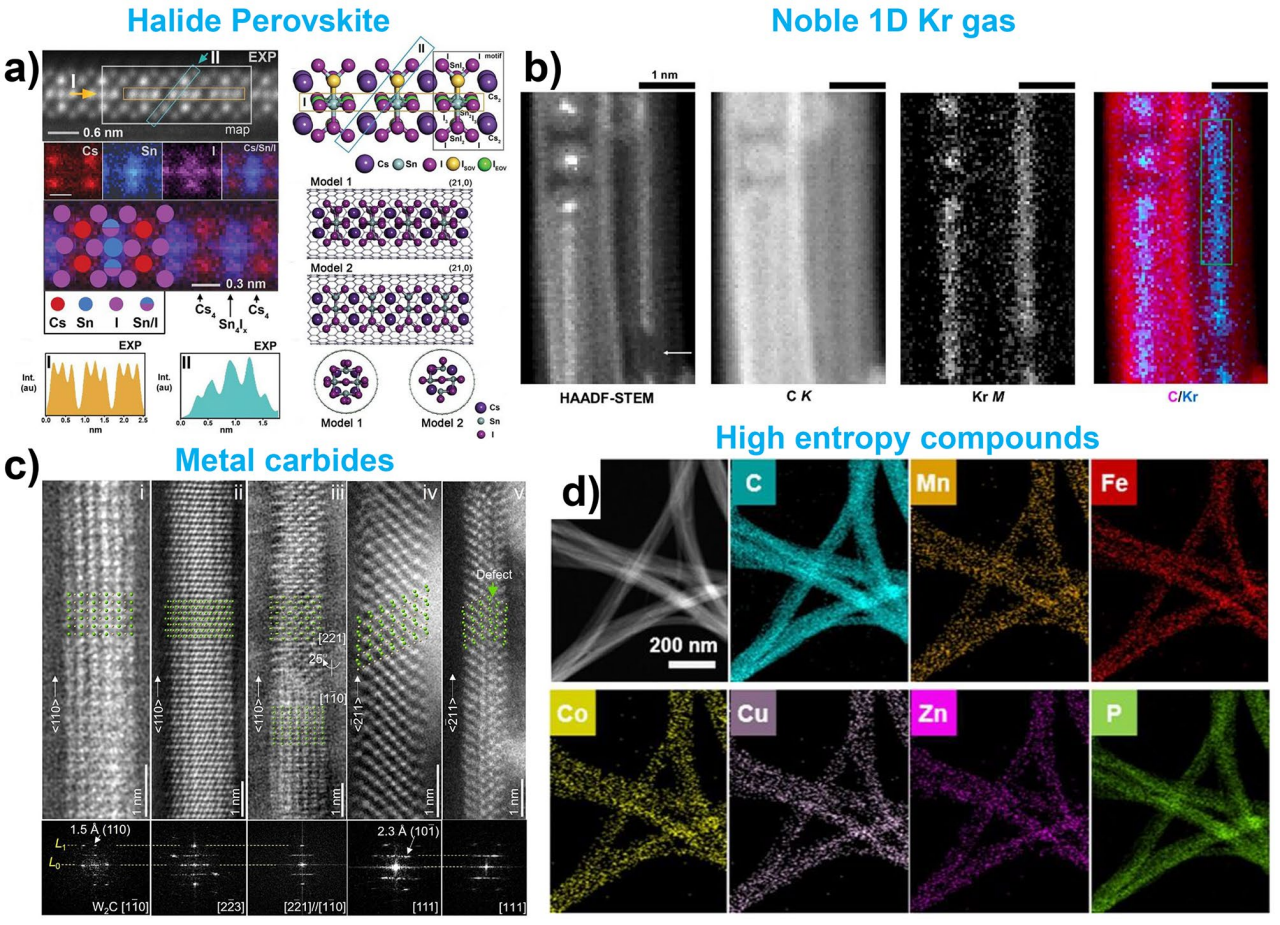
Examples of other materials. (**a**) STEM and EELS map images of perovskite inside CNT with corresponding atomic model. Reproduced with permission from ref. [55], Copyright © 2023, John Wiley and Sons. (**b**) STEM image of Kr gas filled inside nanotube. Reproduced with permission from ref. [87], Copyright © 2024, American Chemical Society. (**c**) STEM images of Tungsten carbide (W_2_C) encapsulation inside a nanotube at different viewing directions. Reproduced with permission from ref. [183], Copyright © 2023, American Chemical Society. (**d**) ADF-STEM image and EDX mapping results of high-entropy compounds encapsulation within the CNT. Reproduced with permission from ref. [97], Copyright © 2024, American Chemical Society

Halide perovskites, known for their sensitivity to environmental factors such as moisture, light, and oxygen, have been successfully encapsulated within nanotubes to improve their stability. Confinement within the nanotubes helps protect these materials from degradation, enabling various structural and dynamic characterizations. Kashtiban et al. successfully synthesized picoperovskites such as CsPbBr_3_ and CsSnI_3_ within nanotubes and characterized their structures using atomic-scale imaging and spectroscopic analysis **(**Fig. 7a**)** [55]. Gao et al. investigated the dynamic motions, including the vibrational, rotational, and translational movements of CsPbI_3_ halide perovskite unit cells, using in situ TEM techniques [54]. Zhu et al. also synthesized CsPbBr_3_ and CsSnI_3_ inside CNTs and demonstrated that the encapsulated structures remained stable for more than 30 days, significantly enhancing their stability compare to their bulk forms [182].

As discussed in the carbon section, the encapsulation of various types of endofullerenes, particularly metal-containing endofullerenes, within CNTs has been explored. Cardillo-Zallo et al. encapsulated Kr@C_60_ within CNTs and modified their structure using e-beam irradiation and thermal annealing [87]. They observed that e-beam irradiation led to local coalescence of C_60_, resulting in the formation of Kr dimers, a phenomenon similar to that observed in previous studies on metal atoms@C_60_. Interestingly, after ex-situ thermal annealing, nested nanotubes formed within the CNTs. In long, well-annealed sections of CNTs, the delocalized Kr atom contrast became visible through STEM imaging and EELS mapping, which confirmed the existence of a 1D, gas-like state of the noble gas within the nanotube **(**Fig. 7b**)** [87].

Metal carbides and high-entropy compounds are formed through a multi-step process, in which metal-containing precursors, such as heteropoly acids and metal chlorides, are pre-filled into nanotubes and subsequently synthesized through further processing [97, 183]. Wang et al. demonstrated the formation of ultra-thin W_2_C and Mo_2_C nanowires confined within CNTs **(**Fig. 7c**)** [183]. These metal carbide nanowires preferentially grew along the < 110 > direction, and exhibited enhanced stability and resistance to H_2_O corrosion, which is consistent with the behavior observed in other materials confined within nanotubes. Du et al. synthesized 1D high-entropy compounds via nanotube encapsulation [97]. They filled the nanotubes with various metal chlorides, which were subsequently heat treated with gaseous phosphorus, sulfur, or selenium to form high-entropy compounds. This multi-step process enabled the formation of 1D high-entropy compounds within the nanotubes, demonstrating the versatility of nanotube confinement for stabilizing complex multi-element systems **(**Fig. 7d**)**.

Thus far, various materials that form unique structures inside nanotubes have been explored. Table 1 summarizes the materials discussed and their structural transformations when confined within the nanotubes.

## Properties of encapsulated materials within nanotubes

As highlighted in the previous sections, nanotube encapsulation enables precise control over the morphology and bonding configurations of encapsulated materials, often leading to novel polymorphs that differ from their bulk counterparts. Various experimental and theoretical studies have explored the electrical, optical, thermal, and mechanical properties of the unique and stable structures of encapsulated materials. This section briefly discusses the unique properties and potential applications of materials encapsulated within nanotubes, both experimentally measured and theoretically predicted [22, 23, 28–30, 37, 38, 41, 48, 49, 60, 61, 82, 86, 179, 187].

**Fig. 8:**
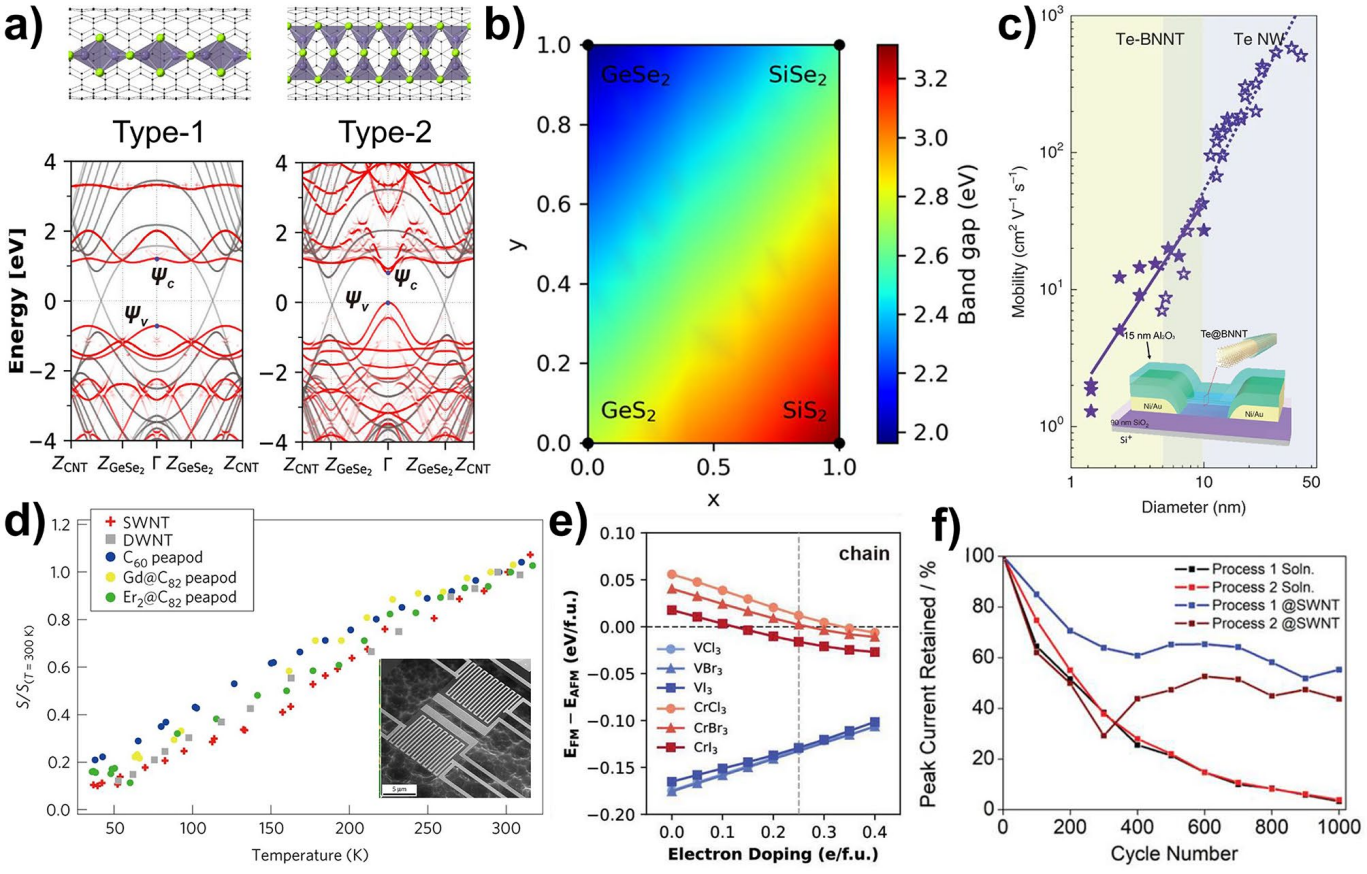
Various properties of encapsulated materials within nanotubes. (**a**) Calculated band structure of single-chain of type-1 and type-2 GeSe_2_ encapsulated within CNTs. Reproduced with permission from ref. [28], Copyright © 2023, American Chemical Society. (**b**) Band gap variation of the type-1 Si_x_Ge_1–x_S_2(1–y)_Se_2y_ alloy chain as a function of composition. Reproduced with permission from ref. [29], Copyright © 2024, American Chemical Society. (**c**) Carrier mobility of Te nanowire encapsulated within BNNT and bare Te nanowire short-channel FETs with various diameters (thickness). Reproduced with permission from ref. [38], Copyright © 2020, Springer Nature. (**d**) Temperature dependence of S of encapsulated CNT bundles and SEM image of measurement device. Reproduced with permission from ref. [22], Copyright © 2017, Springer Nature. (**e**) Calculated magnetic energy of CrX_3_ and VX_3_ single-chains as a function of electron doping. Reproduced with permission from ref. [30], Copyright © 2023, John Wiley and Sons. (**f**) Current retention over 1000 cycle for polyoxometalates molecules (POM) encapsulated inside CNTs and free POM in solution. Reproduced with permission from ref. [61], Copyright © 2019, John Wiley and Sons

As discussed earlier, the tetrahedral structure of GeX_2_ forms 1D chains with different connection modes, depending on the diameter of the nanotube [28]. These structural variations, which are driven by changes in the connection modes, significantly affect their electronic properties. The type-1 structure, which is consisted of edge-sharing tetrahedrons, exhibits an indirect bandgap, whereas the type-2 structure, which is composed of both edge- and corner-sharing tetrahedrons, exhibits a direct bandgap **(**Fig. 8a**)**.

Additionally, encapsulating other materials within nanotubes has revealed fascinating electronic phenomena, such as charge-induced torsional waves and metal-insulator transitions [23, 60]. For instance, in HfTe_3_, as the nanotube diameter decreases and the triple-chain limit is reached, the normally parallel chains spiral together, inducing short-wavelength TAP locking distortion [60]. This structural transformation leads to a metal-to-insulator transition, demonstrating the influence of nanoscale confinement on electronic properties.

Furthermore, controlling the composition ratio of the 1D alloy chains through nanotube encapsulation is a promising approach for achieving a wide range of bandgap turnabilities. Lee et al. showed that 1D quaternary alloy chain, composed of Si, Ge, S, and Se, exhibits wide range tunable bandgaps that range from 1.91 eV to 3.31 eV, depending on their composition ratio **(**Fig. 8b**)** [29]. This tunability provides opportunities for designing materials with tailored electronic properties for specific applications, highlighting the versatility of nanotube encapsulation for fine-tuning the material behavior at the nanoscale level.

The use of encapsulated materials with very narrow nanotubes is a promising approach for developing next-generation electronic devices with thicknesses of less than a few nanometers [11, 45, 46]. Qin et al. encapsulated few-chain and single-chain Te nanowires in BNNTs to study their unique physical properties [38]. In their field-effect transistor (FET) measurement, Te nanowires encapsulated in BNNTs exhibited significantly enhanced current-carrying capability, with a current density of 1.5 × 10^8^ A·cm^− 2^ that exceeded that of most reported semiconductor nanowires. Moreover, encapsulation allowed for scaling down the diameter of Te nanowires to as small as 2 nm, achieving a carrier mobility of 1.85 cm^2^V^− 1^s^− 1^, whereas bare Te nanowire devices are typically limited to a 6 nm diameter **(**Fig. 8c**)**. This excellent performance showed great potential for high-performance FETs with ultrashort channels and in the development of ultimate-scale electronic devices.

The thermoelectric properties of nanotube-encapsulated materials were also investigated. Kodama et al. reported a nanofabrication method to precisely measure the thermal properties of filled CNT [22]. They demonstrated that the encapsulation of C_60_, Gd@C_82_ and Er_2_@C_82_ into the CNT channel significantly suppressed the thermal conductivity(*k*) by 35–55% and enhanced the thermoelectric power(*S*) by approximately 40% compared to pristine CNTs at room temperature **(**Fig. 8d**)**.

The magnetic properties of the materials encapsulated within the nanotubes were also explored. Encapsulated materials, including magnetic metal particles and metal halide compounds, exhibit unique behaviors [30, 35, 36, 111, 185, 188]. Lee et al. synthesized a novel 1D magnetic chain structure by encapsulating Cr and V trihalides (CrX_3_ and VX_3_), representative 2D magnetic materials, within nanotubes [30]. They discovered that the magnetic phases of these encapsulated materials could be modulated through charge transfer interactions with the nanotubes. In the case of CrX_3_, the magnetic phase transition from antiferromagnetic (AFM) to ferromagnetic (FM) occurred as electron doping, whereas VX_3_ remained in the FM phase for all doping ranges **(**Fig. 8e**)**. This finding introduces new possibilities for controlling the 1D magnetism via electrostatic gating.

It has been observed that metallic nanocrystals encapsulated within CNTs can be made to physically and reversibly translate along the axis of the nanotube by application of an electrical current through the nanotube. Remarkably, the position of the nanocrystal can be non-destructively “read out” by measuring the electrical resistance of the nanotube, leading to a nanoscale non-volatile archival memory device [91]. The transport mechanism of the nanocrystal is believed to be a unique electrically-driven nanocrystal deconstruction/reconstruction mechanism [67].

As discussed previously, nanotubes serve as protective layers for encapsulated materials, offering enhanced stability and mitigating issues related to environmental exposure. This advantage makes nanotube-encapsulated materials particularly promising for applications in catalysis and batteries. For example, polyoxometalates (POM) are encapsulated inside CNT to enhance their stability during electrochemical cycling [61]. The encapsulation of POM molecules within the CNTs led to a significantly improved performance compared to that of free POM molecules in solution **(**Fig. 8f**)**. After cycling the POM@CNT materials over 1000 cycles, approximately 50% of the initial current was retained, whereas the free POM molecules in the solution retained only approximately 3% of the current **(**Fig. 8f**)**. Additionally, various other materials, including redox-active molecules, phosphorus structures, and SnS_2_ nanoribbons confined within nanotubes, exhibit improved cycling stability [40, 41, 44, 189]. Moreover, the protective capability of nanotubes, along with their ability to increase the local concentration of the reactant precursors, has found applications in catalysis. Examples include Ru@SWCNTs, Pt/Ru@CNTs, and Fe@MWCNTs, where encapsulation enhances catalytic activity and stability [39, 42, 43]. This demonstrates the versatility and potential of the nanotube-encapsulated systems across for various applications.

## Conclusion and outlooks

In this article, we summarize the recent advances in the encapsulation of various elemental and compound materials within nanotubes, focusing on their unique structures and properties that arise from confinement. The ability to manipulate both the structure and properties of materials at the nanoscale through nanotube encapsulation open new avenues for fundamental research and practical applications. Continued advancements in synthesis techniques, characterization methods, and theoretical modeling are expected to drive the discovery and development of next-generation materials with unprecedented functionalities, ultimately transforming various technological areas.

A wide range of materials, including carbon polymorphs, elemental substances, metal halides, metal chalcogenides, perovskites, metal carbides, high-entropy compounds, and other complex materials, have been successfully encapsulated. The geometrical confinement provided by nanotubes plays a critical role in controlling the atomic arrangement (structure) of encapsulated materials, leading to structures that are not observed in bulk materials.

This confinement effect, combined with the tunability provided by varying the nanotube diameters, enables the formation of nanostructures with tailored properties, such as enhanced electrical, optical, and magnetic behaviors. These properties make nanotube encapsulation a promising technique for advanced applications in electronics, spintronics, quantum devices, and energy storages. Moreover, the ability to control the structure-property relationships through encapsulation provides significant potential for the development of next-generation materials with unprecedented functionalities.

Despite this potential, several challenges remain to be addressed to fully unlock the applications of encapsulated materials in diverse fields. First, ensuring high quality and yield in the encapsulation process is crucial. The filling yield can vary significantly depending on the synthesis parameters such as the nanotube diameter, properties of the filling material, heating/cooling rate, precursor quantity, temperature gradient, and other factors. Among these, the selective use of nanotubes with specific diameters is particularly important. As discussed in this review, the structure and composition of encapsulated materials are highly dependent on the nanotube diameter. To achieve uniform encapsulation, nanotubes with precisely controlled diameters must be used [58, 100, 173, 190]. Existing techniques for sorting nanotubes according to their diameter or chirality should be actively utilized [190–198]. While differences in wettability between CNTs and BNNTs can impact filling yields, BNNT encapsulation has not been as extensively studied; therefore, further research is needed to gain a comprehensive understanding of these effects.

Additionally, an extensive investigation of the properties of materials encapsulated within nanotubes is necessary. Despite the unique electrical, magnetic, and optical properties of encapsulated materials, accurate experimental measurements of properties are challenging. The variability in the encapsulated materials caused by mismatched nanotube diameters and sample preparation issues related to nanotube aggregation are some of the challenges. In particular, the high aggregation behavior of ultra-narrow nanotubes further complicates the fabrication of devices using isolated single nanotubes. Advanced separation techniques for isolating nanotubes with encapsulated materials must be developed to enable precise property measurements and facilitate practical applications. In this regard, encapsulation with chiral-selected or diameter-controlled nanotubes can facilitate property measurements without sample purification issue.

Finally, the development of mass production methods for encapsulated nanotubes is required for commercial applications. Most studies to date have focused on exploring novel structures by filling materials via vapor transport using small amounts of materials. For mass production, solution-based filling methods could present advantages in terms of cost and process efficiency. Further research is required to confirm whether the novel structures obtained via vapor transport can be consistently reproduced through solution-based synthesis. With scalable production, the unique properties of encapsulated nanotubes (high thermal stability, electronic tunability, and magnetic functionality) can be utilized in high-value applications (advanced electronics, battery systems, and electromagnetic shielding composites) where they provide clear advantages. It is expected that development of high-filling ratio, reproducible, and cost-effective filling methods will expand the feasibility of encapsulated nanotubes in a wider range of commercial applications.

## Data Availability

The review is based on the published data and sources of data upon which conclusions have been drawn can be found in the reference list.
